# Best practices for generating and analyzing 16S rRNA amplicon data to track coral microbiome dynamics

**DOI:** 10.3389/fmicb.2022.1007877

**Published:** 2023-02-20

**Authors:** Denise P. Silva, Hannah E. Epstein, Rebecca L. Vega Thurber

**Affiliations:** Department of Microbiology, Oregon State University, Corvallis, OR, United States

**Keywords:** coral, microbiology, methods, high-throughput sequencing (HTS), 16S rRNA amplicon, microbiome

## Abstract

Over the past two decades, researchers have searched for methods to better understand the relationship between coral hosts and their microbiomes. Data on how coral-associated bacteria are involved in their host’s responses to stressors that cause bleaching, disease, and other deleterious effects can elucidate how they may mediate, ameliorate, and exacerbate interactions between the coral and the surrounding environment. At the same time tracking coral bacteria dynamics can reveal previously undiscovered mechanisms of coral resilience, acclimatization, and evolutionary adaptation. Although modern techniques have reduced the cost of conducting high-throughput sequencing of coral microbes, to explore the composition, function, and dynamics of coral-associated bacteria, it is necessary that the entire procedure, from collection to sequencing, and subsequent analysis be carried out in an objective and effective way. Corals represent a difficult host with which to work, and unique steps in the process of microbiome assessment are necessary to avoid inaccuracies or unusable data in microbiome libraries, such as off-target amplification of host sequences. Here, we review, compare and contrast, and recommend methods for sample collection, preservation, and processing (e.g., DNA extraction) pipelines to best generate 16S amplicon libraries with the aim of tracking coral microbiome dynamics. We also discuss some basic quality assurance and general bioinformatic methods to analyze the diversity, composition, and taxonomic profiles of the microbiomes. This review aims to be a generalizable guide for researchers interested in starting and modifying the molecular biology aspects of coral microbiome research, highlighting best practices and tricks of the trade.

## 1. Introduction on coral microbiomes and measures to track their dynamics

Coral-associated microorganisms are critical in the maintenance of animal health, especially in the face of environmental stressors (Bourne et al., 2008; Morrow et al., 2018; Pootakham et al., 2018). The community of these microorganisms (referred to as the coral microbiome) has been identified as a lead indicator of coral health (see Box 1 for more details), with diagnostic signatures that predict coral bleaching, disease, and mortality (Bourne et al., 2016; Zaneveld et al., 2017; Glasl et al., 2019). In the last several years, culture-independent methods for interrogating the coral microbiome have become essential for exploring the impacts of microbiome variability. Specifically, High-Throughput Sequencing (HTS) of 16S ribosomal RNA (rRNA) genes has been widely adopted to understand bacterial and archaeal diversity more generally, making the profiling of existing microbiomes in different host species (Kamke et al., 2010; Ford et al., 2021) a common analysis in the coral field (Siboni et al., 2008; Wada et al., 2016; Ziegler et al., 2017). These studies have advanced our understanding of the role microbes play in coral health, and have produced novel approaches for maintaining or enhancing coral resilience to environmental change, including microbiome engineering (e.g., the manipulation of microorganisms for the benefit of coral health) that can bolster the remediation and protection of corals against rising marine temperatures (Reshef et al., 2006; Peixoto et al., 2017; Epstein et al., 2019; Rosado et al., 2019; Santoro et al., 2021) or contamination from pollutants (Fragoso Ados Santos et al., 2015; Silva et al., 2021).

Box 1: A Primer on Coral Microbiome Research.

Microorganisms are crucial biological components of all living organisms and influence ecological processes (Fraune and Bosch, 2010; Gibbons and Gilbert, 2015). Assessment of their ecology and evolution within hosts has been significantly advanced during the sequencing revolution of the 2000s and today. Due to early adopters (Wegley et al., 2007; Vega Thurber et al., 2009; Littman et al., 2011; Pollock et al., 2011; Sato et al., 2013), corals themselves were a touchstone of using HTS advances in interrogating microbiome features and dynamics within hosts and the environment. As a result, we know a significant amount about coral and reef microbiomes and their dynamics. Since the late 1990s and early 2000s the interrogation of what lives on and in corals, and how they change in response to numerous perturbations, has led to several hypotheses about the role of the myriad members of the coral holobiont.

The coral microbiome is composed of endosymbiotic algae (Symbiodinaceae), microeukaryotes, bacteria, archaea, viruses, fungi, and protozoa (Rosenberg, 2009; Sunagawa et al., 2010; Garren and Azam, 2012; Hernandez-Agreda et al., 2017; van Oppen and Blackall, 2019). Spatially and taxonomically distinct communities colonize all anatomical compartments of corals, which are most commonly split into three for comparative microbial analyses: the surface mucus layer, coral tissue, and coral skeleton (e.g., Sweet et al., 2011; Li et al., 2014; Pollock et al., 2018; Ricci et al., 2022). These compartments each provide unique environmental and physical conditions that can select for specific microbial communities depending on what resources are available (Sweet et al., 2011). Clear differentiation of microbiome structure and function have been found across these three major components in both individual coral species (Bourne and Munn, 2005; Sweet et al., 2011; Li et al., 2014) and among the scleractinian tree of life (32 coral species; Pollock et al., 2018). According to Ricci et al. (2022) the coral microhabitat niche and the phylogenetic characteristics of the host, shape the presence and relative abundance of symbiotic bacterial microorganisms.

Interactions among corals and their microorganisms can be mutualistic, antagonistic, commensal, and competitive. As is true of all symbiosis, these relationships can shift dramatically due to alterations in host or symbiont physiology, the environment, or both (for review see Maher et al., 2022). Bacteria, the most diverse taxonomic and metabolic lineage within the coral microbiome (Rohwer et al., 2001; Rosenberg et al., 2007; Rosenberg, 2009), can serve several essential functions that benefit the holobiont as mutualists, including host protection from pathogens or opportunists (e.g., *via* occlusion and/or antibiotic production; Ritchie, 2006; Bythell and Wild, 2011; Krediet et al, 2013), nutritional supplementation (e.g., vitamins and amino acids; Shinzato et al., 2011) metabolic expansion (e.g., sulfur and nitrogen cycling; Cai et al., 2018; Robbins et al., 2019) and increased growth, survival, and health maintenance through other mechanisms yet untested (Brown and Bythell, 2005; Rädecker et al., 2015; Hartmann et al., 2017; Webster and Reusch, 2017). However, if the coral is environmentally or physically stressed, both resident and/or transient bacteria can become opportunistically pathogenic and cause serious damage, infection, and/or disease (for review see Vega Thurber et al., 2020).

Despite the widespread adoption of HTS methods, the use of culturing methods has not lost its importance in microbiome research. Culture-dependent methods remain crucial for understanding the physiology and metabolism of coral-associated taxa and the interaction between the microbiome, the host, and the environment (see review by Schultz et al., 2022). In addition, culturomics methods can be complementary to HTS, allowing for a complete assessment of the coral-associated microbiome. However, corals are challenging to work with and require additional steps to isolate, extract, generate, and curate microbiome data. Thus, the careful collection, preservation, and processing of coral samples must be optimized to characterize and assess the coral microbiome using different HTS techniques accurately and precisely (Vega Thurber et al., 2022). Thus far, little guidance has been formalized on these best practices in corals due, in part, to the rapid expansion of HTS techniques and the influx of new researchers who aim to conduct them. We envision this article to be a practical guide of well adopted practices for readers who hope to use 16S rRNA gene-based data for conducting analyses of coral microbiomes. Specifically, we describe (1) strategies for collection, preservation, and processing samples to assess the different compartments of coral; (2) comparative extraction methods to isolate and preserve microbial DNA; (3) strategies to avoid host and off-target contamination during PCR and HTS library construction; (4) common approaches and issues surrounding different sequencing platforms; (5) basic quality control, analytical pipelines, and software that can be used to access some measures of coral microbiome diversity, composition, and stability.

## 2. Use of high-throughput sequencing in tracking microbiome dyamics

Due to the limitations of culture-based methods, culture-independent techniques have progressed overwhelmingly in the past 20 years. Early studies utilizing culture-independent methods relied on low-throughput sequencing technology (e.g., Sanger sequencing) and finger-printing methods (e.g., TRFLP and DGGE) that led to many foundational inferences about the coral microbiome (Rohwer et al., 2002; Klaus et al., 2005; Sunagawa et al., 2010). Because these techniques result in relatively low numbers of sequences for fairly high costs, along with issues in poorly curated databases for comparative analysis, these techniques fell out of favor as HTS gained traction. The major advantages of HTS methods are the (1) high yield of data resulting from millions to billions of sequencing reads in a single run, (2) the low cost per base, and (3) comparable genetic data for cross-system compatibility due to wide adoption across the microbial ecology field. With HTS technology, it is possible to design and implement experiments with many more samples and replication (i.e., increased statistical power) that can provide advantages when assessing changes in microbial composition of different coral species and exploring spatiotemporal variability (Haydon et al., 2022) while decreasing the likelihood of type I and type II errors.

Currently, the most used microbiome HTS methods is amplicon sequencing, or the amplification of a single or multiple gene sequences [e.g., 16S rRNA and *recA, gryA* genes and the internal transcribed spacer (ITS) region]. Most of these target genes are present across specific domains or clades of organisms, and many are well studied molecular clocks that are useful for phylogenetics (Yang et al., 2016). HTS technology has itself evolved with many iterations and platforms including: 454 pyrosequencing (Margulies et al., 2005), Ion Torrent PGM (Personal Genome machine; Rothberg et al., 2011), PacBio (Pacific Biosciences; Eid et al., 2009), MinIon (nanopore sequencing; Mikheyev and Tin, 2014), and Illumina (Bentley et al., 2008). Over the past 5 years, Illumina Miseq and Hiseq platforms have been the most widely used although the HiSeq platform is now being decommissioned and replaced by the Illumina NextSeq and NovaSeq platforms.

These sequencers can produce single reads of varying lengths, and platforms can also generate linked or ‘paired end reads’ that represent the forward and reverse portions of longer amplicons that may not reach across the sequenced reads. Along with length, importantly, the number of resulting reads also can vary significantly. For example, where NextSeq 550 has generated between 260 and 800 million reads, and MiSeq (with the Reagent Kit V3) can currently result in 30–40 million read pairs. Other platforms in place of the HiSeq, such as NextSeq 1,000 and 2000, where the maximum read length is 2×300 bp, can generate between 100 million–2.4 billion reads as of December 2022. Higher read depth can increase clustering and longer read length can increase the frequency of accurate taxonomic calls using reference libraries (see data analytics section below). Currently, MiSeq is commonly used for amplicon sequencing due to its long read length (2 × 300 bp), low cost, and high accuracy. However, the newest and most advanced Illumina sequencing platform, NovaSeq, is capable of generating up to 40 billion paired-end reads (reads lengths up to 2 × 250 bp) at a low cost. Thus, NovaSeq can be used for large-scale projects. Therefore, the optimal platform will depend on the nature and the objective of the study. Further, sequencing platforms are a rapidly evolving technology, and we encourage readers to compare platforms to inform such decisions (e.g., Singer et al., 2019).

### 2.1. The increasing number of options for the use of HTS in coral microbiome analysis

Due to the increase in the diversity of genetic tools and analysis pipelines, many researchers entering the coral microbiology field struggle to determine the best and most adopted techniques to answer specific questions. At the same time, methods rapidly change, with new techniques constantly pushing the boundaries of what we can do with HTS data. New concepts in how samples should be processed and analyzed are constantly changing. Thus, in addition to the experimental design considerations, we must consider factors that may influence the choice and application of both processing and analytical methods to elucidate different aspects of coral microbiology.

Identifying the best sample processing methods and molecular techniques to apply in a study can be laborious, and manuscripts can suffer in peer-review if the methods are not up-to-date or fully benchmarked. That said, no method is a panacea, and appropriate methods of collection, preservation, processing, and molecular tools must be tailored depending on the focus or question of the study, the source of materials, and the samples’ history and provenance. For example, each coral species, individual, or even compartment may require optimization of techniques. These study-specific details will matter when designing and optimizing the HTS approach, but fundamentally the steps are similar: collect the sample, extract microbial DNA, amplify the target gene using PCR, generate sequencing libraries, and finally *in silico* analysis of microbiome communities.

### 2.2. Choosing in-house HTS library preparation vs. commercial or institutional sequence providers

There are now several companies and university core facilities that will conduct many or all of the below steps as paid services. Often the services are itemized and can be adjusted and personalized to best suit any one project. With economies of scale this can be an affordable, standardized, and reliable means to get samples and data back quickly. Both approaches have different benefits, and whether one chooses to conduct the work in-house or through such a provider is entirely up to the researcher’s needs, goals, and finances. For example, DNA extraction and HTS library preparation services can reduce time and/or financial costs and lower levels of contamination due to the use of robotic preparations. However, the ‘black box’ nature of these providers makes scientific transparency difficult and reduces the opportunity for students/researchers to learn the process. For truly comparable datasets, the methods conducted by a service team or set of researchers must be as identical in their protocols as possible, making transparency critical. Even small deviations from any of the major steps of the process can cause extraction, amplification, and sequencing biases that may be revealed in downstream microbiome analyses as differences in taxonomy and composition (see below).

## 3. Best practices and options in methods for assessment of coral microbiome features

In the following sections, we describe several considerations and methodologies for generating accurate and precise assessments of coral microbiomes, which have unique requirements compared to many other host-associated microbiomes. We envision this as a ‘how-to-guide,’ but recognize that not all methods will be suitable for every study. Adoption of the methodologies below should always be considered and adapted in light of each research group’s specific questions, system needs, and available resources.

### 3.1. Collection, preservation, and processing methods

Stony corals have only 2 tissue layers (gastroderm and ectoderm), a mesoglea, and an aragonite skeleton (Muscatine, 1969; Grottoli, 2001). Many corals produce a mucus coat for protection from environmental shifts and potential disease-causing agents (Allen, 1983; Shnit-Orland and Kushmaro, 2009). Differences in microbial assemblages between coral compartments (Sweet et al., 2011; Li et al., 2014; Pollock et al., 2018) and the functional role each plays makes the selection, proper sampling and processing of these compartments critical to research outcomes.

**Figure 1:**
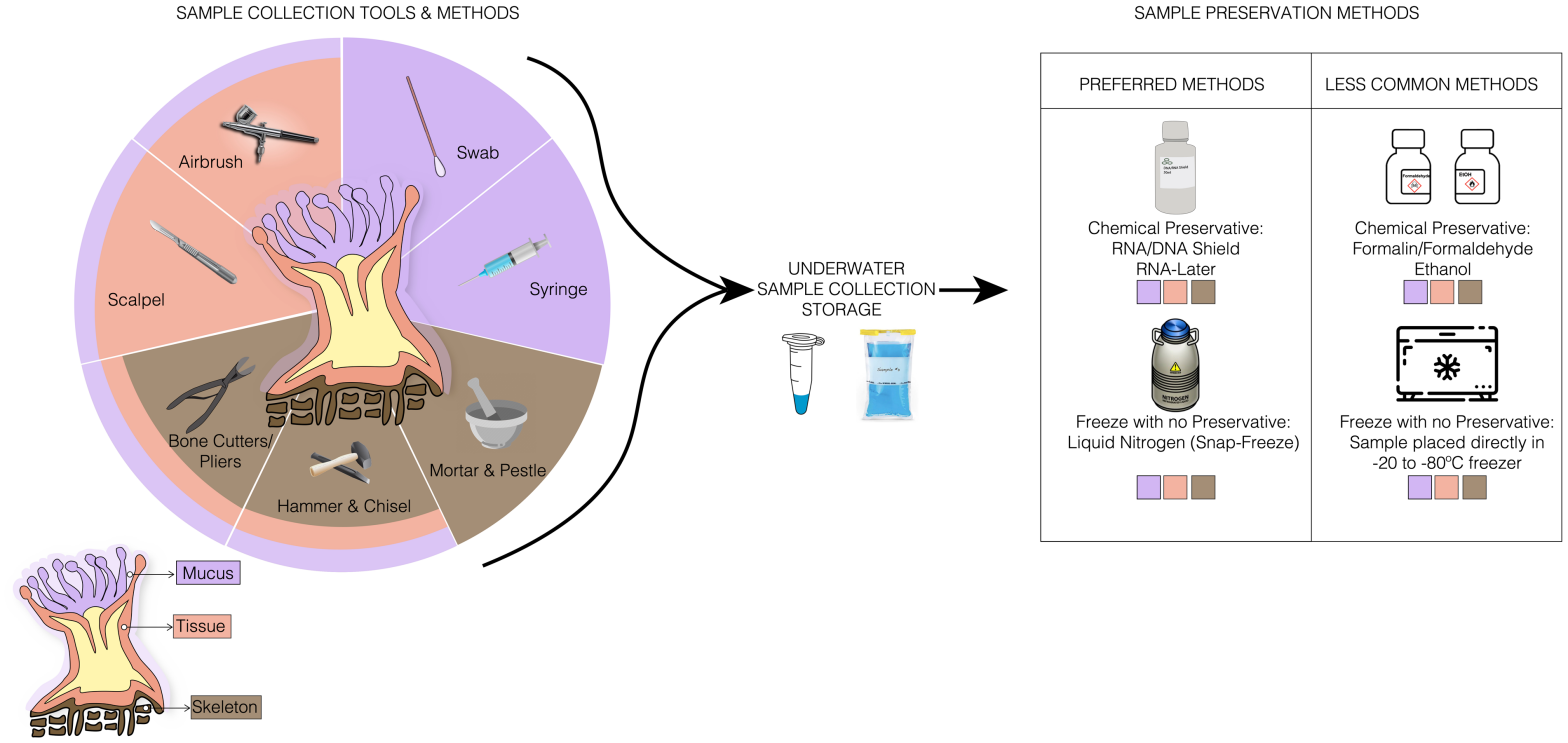
Illustration of ideal sampling strategies for each coral compartment, collection storage, and the preferred and less common sample preservation methods used for any coral samples. In samples collection methods, there are specific tools for a compartment of the coral, such as the syringe used only for the collection of mucus, and tools, such as the bone cutter, used to collect any sample from the corals.

Several collection methods and/or sampling techniques can be applied to each coral compartment of interest (Figure 1). These sampling techniques can vary by their invasiveness and potential for negative effects on the holobiont; a consideration that must be ethically and experimentally weighed by the researcher before sampling begins. For example, removal of large portions of a colony can alter host physiology and/or cause extensive damage that may lead to colony mortality. Adjusting sampling to simultaneously reduce negative consequences on the colony or individual while also accurately analyzing the specimen for microbiome features is critical. Below, we describe available methods for sampling, preserving, and processing coral samples for microbiome work and briefly discuss when to use each.

Whole coral specimen – Small fragments (can range between 1 and 8 cm^2^) of corals can be used for microbiome research evaluating the dynamics of the holobiont. Experiments or surveys might require repeated sampling of individuals over time or the complete removal of a specimen during experimentation, so it is important to consider size at the beginning of your sampling period. For 16S amplicon analysis, only a very small fragment is required for accurate microbiome characterization, and amplicons can be generated from as little as 2 mm of diameter. However, to our knowledge there have been no studies that systematically examined the efficacy of DNA extraction from different fragment sizes. Typically, if other downstream analyses (e.g., other ‘omics or physiological analyses) are conducted alongside 16S analysis, specimen sizes may need to be much larger, depending on the analyses of interest and the size and shape of the colony. During sampling, scleractinian corals are often collected using a hammer and chisel for massive or very thick branched corals, or bone cutters and snips for more delicate and smaller branching or plating corals (Apprill et al., 2016; Neave et al., 2017; Roitman et al., 2020). In the case of branching corals, collections can be made using needle-nose pliers. Underwater work makes it extremely difficult to maintain sterile technique, but wearing gloves and changing tools between sampling, especially if investigating disease, should be done to avoid cross-contamination among samples. See Box 2 for details on PCR contamination and mitigation strategies).

After collection, the specimen should be stored in sterile tubes or together with seawater in sterile, hermetically sealed bags, such as Whirl-Pak bags (Nasco, Salida, CA, United States) filled with local seawater (Kellogg et al., 2016; Neave et al., 2017). These bags are recommended because they are durable, leak-resistant, freezer-safe, and can be ordered with “write-on” labels that ensure permanent pen will not rub off in the freezer.

Box 2: PCR contamination.

PCR is a highly efficient, processive, and relatively insensitive molecular process. Although these are typically advantageous attributes, there can be downsides that require us to use excessive precautions to avoid contamination from exogenous sources. Contamination can occur between and among samples (cross-contamination) or from an exogenous DNA source. Cross-contamination can be due to the mishandling of samples and/or materials as well as imprecise sterile technique. For example, practices such as keeping sample tubes open during PCR setup, pipetting reagents quickly that can generate aerosols, or inadequate disposal of tips and tubes can contaminate nearby samples and surfaces which can cause future contamination. The exogenous contamination source is related to the improper handling and storage of PCR reagents (e.g., primers, Taq polymerase, and water) and contaminated working environment. For these reasons, if at all possible, reagents should never be stored together with DNA samples or amplicons. The use of non-sterile materials such as pipettes, tubes, tips, laminar flow hood, and the incorrect or inappropriate use of PPE such as non-sterile gloves can also introduce exogenous DNA. To control for contamination, it is now standard to conduct, and sequence replicate negative control PCRs. In the event of library contamination, in silico removal of the sequences in the negative control libraries will improve the accuracy of the study.

Once introduced into a lab or system, PCR products or exogenous DNA can lead to a cascade of contamination throughout the laboratory, making it difficult to reestablish sterility. Therefore, preventing and if necessary, removing DNA contamination is a significant challenge that must be done effectively as contaminants can remain on surfaces for an extended period. For this reason, numerous methods of decontaminating DNA from laboratory surfaces have been developed including UV radiation. The UV radiation of the laminar flow hood and autoclave (Gefrides et al., 2010; Ziubrii, 2019), enzymatic method with exonuclease III (Zhu et al., 1991), use of Uracil-N-glycosylase (Longo et al., 1990), endonucleases (DNAse; Eshleman and Smith, 2001; Klaschik et al., 2002) and chemical methods such as hydroxylamine and hydrochloride (Aslanzadeh, 1993).

The most used methods are UV radiation, alcohols (ethyl alcohol and isopropyl alcohol), sodium hypochlorite, and DNase treatment. UV radiation damages the double strand of DNA, forming products, such as pyrimidine-pyrimidine and cyclobutyl pyrimidine dimers that impede the action of Taq polymerase (Cadet et al., 1986; Giussani et al., 2018). The efficiency of UV radiation will depend on the distance from the decontaminated surface, the molecular weight of the DNA, and exposure time. It is recommended that the UV radiation decontamination process take approximately 15 min. PCR reaction reagents are sensitive to UV radiation. Therefore, it is not recommended to add reagents to the laminar flow while the UV light is on because this practice can affect the amplification of the DNA of interest. Alcohols such as ethyl and isopropyl alcohol help precipitate DNA, but they also denature proteins and inhibit enzymatic reaction when diluted (Wu et al., 2018b). Alcohols can be used for surface decontamination in concentrations between 60 and 70%. The most used is ethyl alcohol. However, there is no difference in effectiveness between the two. Pure sodium hypochlorite at a concentration between 1.0–1.5% is also widely used as a surface decontaminant (Fischer et al., 2016). This reactant can damage the cell membrane, inhibit enzymatic reactions, and directly damage the carbon-hydrogen bonds of DNA through oxidative cleavage (Prince and Andrus, 1992; Kampmann et al., 2017). Commercially-available sodium hypochlorite (bleach solution) in concentrations between 5.25–6.15% can also be used but should be used at a concentration of 10% (sodium hypochlorite 0.5–1%; Goodyear, 2012). When using sodium hypochlorite on the surface, or a 10% bleach solution, wait 10 min and then remove excess bleach with ultrapure water or DNase, as prolonged use of hypochlorite can cause corrosion to laboratory surfaces.

DNase treatments have also been widely used on equipment and surfaces without the risk of material degradation. The most commercially used solutions are DNA away (Thermo Scientific, Wilmington, DE), and DNAzap (Invitrogen Corp., Carlsbad, CA). Even with several decontaminant options, these techniques may not eliminate all contamination. It is difficult to carry out the decontamination of DNA molecules with a low molecular weight (less than 200 bp). Therefore, it is recommended the combination of techniques to have efficiency in the sterilization process. Champlot et al. (2010) combined strategies, such as UV radiation and DNAse treatment. For surface and equipment decontamination combined methods such as 75% ethyl alcohol, UV light, and hypochlorite solution ca be used (Wu et al., 2018b).

A major concern for coral microbiome research is that the PCR process is highly susceptible to contamination, leading to significant accuracy problems downstream. This is because a single PCR can produce thousands of amplifiable DNA molecules even if extremely rare in a sample. Thus, any foreign DNA can be amplified and contaminate your PCR and your resulting microbiome library. To ensure sterility during the pre-PCR process, wear clean gloves and, where possible, prepare PCR reactions inside sterile or laminar flow hoods. Use sterile tubes, tips, and keep all materials inside the hood decontaminated with 70% ethanol or bleach 10% and UV light for 15 min (Aslanzadeh, 2004). Use sterile tips, pipettes, tubes, and racks exclusively stored inside the PCR hood and always use DNA-free reagents. All PCR reagents should be reviewed regularly and exchanged for new stock reagents if contaminated. It is also recommended to use special care when making stocks and then aliquot ‘working stock’ small volumes of reagents to ensure no new contamination of expensive and hard to replace highly concentrated stocks. Further post-PCR amplicon libraries should be stored safely and, if possible, never returned to the site of pre-PCR steps as they can contaminate all your materials and future studies. As a result of this well-known issue (Fox et al., 1991; Roux, 1995; Scherczinger et al., 1999), every lab should treat PCR products as a potential source of contamination. To avoid this, many labs separate the physical PCR setup phase from the actual amplification stage (Aslanzadeh, 2004). We suggest that, if at all possible, materials used for PCR setup are designated to a biological safety cabinet that has full UV decontamination capabilities and all PCR amplification steps, and all resulting PCR products and materials are kept in a separate room.

Contamination can also occur during the process steps that precede PCR, such as *via* DNA extraction kits (Salter et al., 2014; Weiss et al., 2014) Known as the ‘kitome’, contamination of DNA extraction kits can occur during the processing and preparation of kit reagents. Bacterial components from contamination may vary between kits (see Salter et al., 2014), and removal of contaminants can be difficult. Therefore, it is essential to use extraction and PCR negative controls (blanks) in parallel with real samples throughout the process. Sequencing negative controls from each stage of the extraction and PCR can help to identify specific contaminating bacterial taxa or sequences that arise erroneously and provides a confirmation that the coral microbiome profile is accurate.

After collection, it is necessary to rapidly remove any excess liquid and place samples in as cold of conditions as possible (ideally ultra-frozen in liquid nitrogen), within preservatives (e.g., DNA/RNA Shield, RNAlater, or salt buffered DMSO), or fixatives (e.g., aldehydes) to prevent the microbiome from changing in composition, total abundance, and function (Gaither et al., 2011; Hernandez-Agreda et al., 2018b; Gardner et al., 2019; Pratte and Kellogg, 2021). The ultra-freezing method is widely used, as it preserves the sample instantly and leaves it free of artifacts present in chemical preservatives (Vega Thurber et al., 2022). However, this method may not be readily accessible under field conditions. Thus, the other methods available to preserve the integrity of microbial DNA or RNA include DNA/RNA shield (Zymo Research Corporation, Irvine, CA), which can stabilize nucleic acids at room temperature for up to 24 h (after which they must be placed in fridge or freezer according to the manufacturer’s guidelines), and RNAlater at 4°C overnight to allow the buffer to infiltrate the samples before being transferred to −20°C or −80°C (Kellogg et al., 2016; Carradec et al., 2021). However, the efficiency of the preservation method depends on the next steps in the nucleic acid extraction methods. In the case of RNAlater, DNA extraction methods based on alcohol exclusion steps are not ideal because the high concentration of salt that is present in this solution can precipitate along with the DNA and can further inhibit later steps in the protocol (Athanasio et al., 2016). The use of different stabilizers might also limit what downstream kits can be used. For example, RNA/DNA Shield is highly compatible with its manufacturer’s extraction protocols but is not optimized for other kit-based extraction methods. Always consult with the manufacturer when adapting sampling steps that may necessitate alterations to downstream molecular biology processes.

For post sample processing, fragments of the whole coral are usually either subsampled and/or placed directly into sterile tubes or tubes from DNA extraction kits that contain preservatives, macerated using a mortar and pestle while keeping the sample dry and cold with liquid nitrogen (Santos et al., 2012; Li et al., 2013; Zhang et al., 2015; Kwong et al., 2019), or homogenized using a bead beater (e.g., FastPrep24, MP Biomedicals, Irvine, CA; Klaus et al., 2005; Sekar et al., 2009; Sato et al., 2013; Kellogg et al., 2016; Biagi et al., 2020). Each of these methods can result in enough high-quality material for 16S amplicon library generation.

Mucus – Coral mucus can be used to investigate the role of the microbial assemblage and the interactions between coral and environment. Many of the initial experiments on corals used mucus as a way to track microbiomes overtime without extensive damage to the host and have been used as a diagnostic tool for coral health (Carlos et al., 2013; Glasl et al., 2018). However, mucus sampling tends to result in more variable assemblages of microbes as these communities tend to have more transient microbiome members. It is important to note that both the amount produced and the age of the mucus can have major impacts on microbiome community composition (Glasl et al., 2016), which may limit the comparative power of this technique.

Mucus collection is typically carried out underwater using a sterile syringe (without the needle) and negative pressure. Sometimes minor abrasion is necessary to induce the coral to generate mucus (Hadaidi et al., 2017). The mucus is aspirated carefully from the coral surface without causing excessive damage and immediately after collection, the syringe can be inverted, allowing the mucus to accumulate at the base of the syringe due to its higher density. Ideally as much of the excess seawater should be expelled prior to transporting and/or transferring the mucus. Mucus can also be collected by sterile swab that is rolled or slid along the coral surface lightly collecting visible mucus *via* adhesion (Engelen et al., 2018; Weiler et al., 2018). This exposure method, in principle, reduces seawater contamination but is complicated by removing the animal from its natural environment and the unreliability of all coral species to produce mucus in this way.

Once collected, mucus can be transferred from the syringe to sterile tubes and be quickly placed on ice or dry ice, frozen in liquid nitrogen, transported to the laboratory and stored at −20°C or −80°C, or placed in stabilizing buffers or preservatives (see section above) until DNA extraction.

Tissues – Coral tissue is a primary target for evaluating coral microbiome structure, function, and evolution. Given the intimate nature of hosting intra-and extracellular microbes in the tissue, the physiological and evolutionary interpretation of changes in coral tissue microbiomes are generally more straightforward than mucus-associated microbiomes, which are more variable and highly influenced by the external environment (Pollock et al., 2018).

To collect tissue samples, a coral fragment is usually collected as reported above for the whole coral specimen and then fractionated using a variety of methods that remove the tissue from the skeleton, such as airbrushing or water-picking with sterile fluids like phosphate buffered saline (PBS) or 0.22 μm filtered seawater. The use of PBS is a particularly effective strategy since this solution is cheap, isotonic, can come in sterile forms, and can be diluted with samples without generally interfering with any downstream molecular biology or chemistry in the samples (Hester et al., 2016; Weber et al., 2017). Tissues can also be dissected from the skeleton with a scalpel or razor blade, although the skeleton would almost certainly be present in any sample using this method (Littman et al., 2010; Kvennefors et al., 2012; Sudek et al., 2012). Another means to acquire exclusively tissue would be to add preservative and/or fixative that would allow downstream DNA extraction and then decalcify the coral using salt buffers or a mixture of formic acid and sodium nitrate (Berzins et al., 2011; Hernandez-Agreda et al., 2018a; Bergman et al., 2022). Tissue samples can be stored in ultra-freezers, in 100% molecular grade ethanol, or depending on the subsequent microbiological analysis, it can be fixed with 4% paraformaldehyde at 4°C for 12 h (Staley et al., 2017; Hernandez-Agreda et al., 2018a). In addition, the preservation of the tissues can be done with liquid nitrogen or salt-saturated dimethyl sulfoxide [salt-saturated DMSO (Gaither et al., 2011; Zhang et al., 2015)]. These substances are recommended for distant collections where options, such as freezers are unavailable, as salt-saturated DMSO and liquid nitrogen can remain viable for a long time and still result in accurate 16S library generation.

Skeleton – Although typically thought of as acellular, the skeleton contains a diverse and interesting collection of microbes. Collection and preservation methods can be done in the same way as with whole fragments but with additional steps to remove the mucus and tissues. During processing the skeleton can be separated from mucus and tissue through airbrushing with a sterile solution as discussed above (Neave et al., 2017; Weber et al., 2017; Marchioro et al., 2020). After this procedure, the skeleton samples can be preserved in liquid nitrogen and/or macerated with a sterilized mortar and pestle. Although we know of no papers that discuss this, it is also likely that bleached and/or dried coral specimens may contain internal DNA that could be used for coral microbiome studies. Future investigations on benchmarking such methods are necessary.

### 3.2. Nucleic acid extractions for coral microbiome analysis

The generation of 16S amplicons to track coral microbiomes requires efficient DNA extraction of both bacterial and host cells. Extraction protocols include three main steps: cell lysis (also called cell disruption or cell digestion), precipitation, and purification. Whether using a commercially available kit or an in-house method, these three steps are necessary for effective and high-quality DNA extractions. While it is difficult to standardize the extraction process to a single method due to the diversity of coral species and different sample types (e.g., coral compartment), several methods are commonly used that rely on readily available DNA extraction kits with different protocols (see below). Kits optimized for soil microbe samples are often good choices for coral DNA extractions because, like corals, soils contain high levels of inhibiting compounds, such as humic matter, that require additional DNA purification steps, making these kits more thorough in eliminating biological inhibitors.

The first crucial step in coral microbiome extraction is cell lysis, which is used to make microbial DNA accessible (Santos et al., 2012). In coral tissues, lysis can be challenging due to the presence of the mesoglea, a gelatinous layer between the epidermis and gastrodermis that is rich in collagen fibers and that are difficult to break, impeding to access to the microbial community contained within internal tissue. Without adequate cell lysis, extracted DNA may not accurately represent the microbial community. While some kits come with mechanical lysis tubes included (e.g., Qiagen PowerSoil, ZymoBiomics, etc.), the size and type of lysing matrix (often made from garnet, zirconia/silica, and/or glass beads) can affect both the efficiency of lysis and the amount of microbial DNA obtained. According to Weber et al. (2017), smaller beads may target the smaller microbial cells, whereas larger beads can also lyse eukaryotic cells in the coral and produce a flood of eukaryotic DNA in the sample. To account for variations in cell size, you can also use a combination of different types of beads, such as Lysing Matrix “A” bead-beating tubes (MP Biomedicals, LLC, Santa Ana, CA, United States) which combine garnets with large 1/4-inch ceramic spheres. These lysing matrices can be added to preservative collection tubes to stabilize nucleic acids and prepare for mechanical lysis at the same time. Mechanical lysis (aka “bead-beating”) can be performed using commercial bead-beaters, such as the FastPrep24 (MP Biomedicals, Irvine, CA), the PowerLyzer24 Homogenizer (Qiagen Inc., Valencia, CA, EUA), or simply using a vortexer.

For effective breakdown of cells, most DNA extraction kits and protocols also use a chemical lysis, which is performed with a buffer that contains either an ionic detergent such as sodium dodecyl sulfate (SDS), which solubilizes, denatures, and breaks down cell membrane proteins to release DNA (Brown and Audet, 2008), or an enzyme. One of the enzymes used to lyse bacterial cells is lysozyme, which breaks down the glycosidic bonds in bacterial cell walls (i.e., the peptidoglycan layer; Shehadul Islam et al., 2017). In coral microbiome studies, it may be useful to use more than one type of chemical lysis to ensure the lysis of both gram-positive and gram-negative bacterial cells occurs, as gram-negative bacteria contain an outer membrane that can prevent lysozyme from accessing the peptidoglycan cell wall (Salazar and Asenjo, 2007; Ketchum et al., 2018). For example, enzymes such as proteinase K can be applied in an incubated digestion step (37–70°C) to increase yield and inactivate nucleases that could degrade DNA or RNA during the purification process. Proteinase K, when combined with chemicals such as SDS, Ethylenediaminetetraacetic acid (EDTA), enzymes such as RNAse, trypsin, and others, can improve DNA cleaning efficiency (Banaszak, 2007). Given the possibilities of combining methods, mechanical and chemical cell lysis can be optimized according to the specificity of the sample and the DNA to be extracted. However, it is important to note that some methods of cell lysis can increase PCR interferences due to the disruption of eukaryotic cells whose chemical composition (e.g., humic acid in tissues and calcium ions in skeleton) may affect the quality and quantity of bacterial and archaeal DNA and its amplification through inhibition of chemical reactions (Lorenz, 2012).

Following lysis of microbial cells, DNA is precipitated to separate it from cell debris and off target macromolecules. Alcohol (isopropanol or ethanol) and salt solutions are typically used to make the DNA insoluble. After precipitating the DNA and eliminating cellular debris, purification is conducted, again using alcohol as its main agent. Until recently, the conventional DNA extraction technique was called phenol: chloroform:Isoamyl alcohol method. This approach can be a cheap and efficient option, but any residual phenol can contaminate samples and make them difficult to work with downstream. Furthermore, these methods use caustic and volatile compounds (i.e., phenol) and must be carried out inside a chemical safety cabinet. Advancements in commercial kits have reduced reliance on this technique.

The effectiveness and accuracy of recovering high-quality and purity DNA can vary according to the extraction kits (Galkiewicz and Kellogg, 2008; Weber et al., 2017). A variety of kits have been used for the extraction of DNA from different coral species and different parts of the coral (Rodriguez-Lanetty et al., 2013; Glasl et al., 2019; Weber, 2020). For example, Santos et al. (2012) tested the efficiency of DNA extraction from fragments of the *Mussismilia hispida* coral by comparing 4 different DNA extraction kits (ZR Soil Microbe DNA Kit, Zymo Research, PowerSoil DNA Isolation Kit, FastDNA SPIN Kit for soil). They showed that the FastDNA SPIN Kit for soil was more efficient for high DNA yield compared with the other kits. Also, Weber et al. (2017) carried out a study comparing other DNA extraction kits from MoBio laboratories (PowerSoil^®^, PowerPlant^®^ Pro, PowerBiofilm^®^, and UltraClean^®^ Tissue & Cells) in 7 different species of coral and evaluated the amplification efficiency of 16S rRNA. PowerBiofilm^®^ produced higher DNA yield and a more diverse microbial community when compared with other kits. These studies suggest that no specific DNA extraction kit must be used with coral samples (as exists for soils and plants studies). Bergman et al. (2022) also compared the output of microbial community analysis from 2 different coral species with 3 different kits (see citation for details) and found that, at least for the same coral species, each kit resulted in similar alpha and beta diversity estimates. Given these data, we recommend that research be carried out on the lysis and methods that each DNA extraction kit uses to determine the most suitable kit for a given sample type.

### 3.3. Amplicon sequence amplification

Amplification of microbial DNA sequences to create ‘amplicons’ is conducted *via* PCR. Each reaction consists of a mastermix that includes Taq polymerase, magnesium ions, free deoxynucleotide triphosphates (dNTPs), primers for an especific target gene region and DNA template. Below we discuss the steps of PCR and the considerations for choosing Taq polymerases and primers that will ensure effective amplification.

Primers – Primers should be selected to cover the ends of the specific rRNA gene region of interest, such as the forward primer that attaches in the 3′ → 5′ direction (the antisense strand) and the reverse primer that attaches to the last nucleotide of the region to be amplified in the sense 5′ → 3, direction (the sense strand). Primers are commercially synthesized and generally have a size of around 15–30 nucleotides with guanine-cytosine (G and C, respectively) content that can range between 40 and 60% (Lorenz, 2012). Furthermore, it is recommended that the primers have a CG clip at their ends, that is, the presence of C or G in one of the last 5 sequences to ensure primer binding to the complementary sequence.

While several target genes can be used for microbial taxonomic analysis, such as 23S rRNA (Pei et al., 2009), *rpoB* (Ogier et al., 2019), and others, typically, the 16S rRNA gene is used due to its presence across all bacterial and archaeal lineages and its slow evolutionary rate of change. The 16S rRNA gene makes up one component of the small subunit of the bacterial ribosome and is highly conserved due to its essential function of aligning mRNA to the ribosome for accurate and processive protein production. Interspersed with conserved regions of this gene are highly variable regions (V1-V9), which provide smaller, unique sections of gene sequence for comparison (Caporaso et al., 2011; Bukin et al., 2019). Which variable region to use for amplicon sequencing is hotly debated in the field, and the choice of primers is an extremely important consideration for any study. According to Kim et al. (2011), different regions of the 16S rRNA gene can produce different results regarding species richness and diversity of the microbial community. Primers that target the V4 region of 16S rRNA, in particular 515F and 806R and 806Rb, are currently the most commonly used for analyzing the taxonomic diversity of Bacteria and Archaea in corals (Apprill et al., 2015; Walters et al., 2016). Although widely used, this primer set has considerable downsides for the study of coral microbiomes (see section on host off-target contamination) and caution must be taken when using this popular primer set.

While performing PCR, some problems associated with the use of any primer set may arise. For instance, the creation of “primer-dimers” occurs during the annealing process where the primers may anneal with each other rather than the template DNA. This annealing occurs because primers are complementary and can bind at the 3’ end. This failure can be seen in agarose gel electrophoresis images as intensely illuminating low molecular weight bands (<100 bp). For this problem, Lorenz (2012) suggests optimizing the amount of primer for the amount of template DNA in the reaction, although dimers can also be removed during cleaning steps.

A primer pair is considered ideal during amplification when they can achieve amplification efficiency and specificity, maximize coverage of the microbial community, and minimize PCR bias (Sambo et al., 2018). These optimal characteristics are attributed to (1) the position of the nucleotides compatible with the template DNA, avoiding amplifying other target sequences that are not selected; (2) amount of nucleotides in the primer; (3) GC (guanine-cytosine) content which should contain about <60% so that it does not interfere with successful amplification (> 60% tends to increase hydrogen bonds between GC and generate secondary structures such as hairpins and formation of dimers; Assal and Lin, 2021); (4) avoid sequences with dinucleotides (such as CGCGCG or ATATAT) so that there is no formation of secondary structures; (5) use of primers or degenerate primers to minimize PCR bias.

The efficiency of target gene amplification can be compromised and generate PCR artifacts as well. These artifacts can result from errors such as chimera formation during amplification or uneven distribution of PCR product amplification, also called “PCR bias” (Acinas et al., 2005). PCR bias can be attributed to primer incompatibility with some targets that can occur even for a single base. Thus, to avoid bias and cover the community of interest, primers can be modified using nucleotide sequences corresponding to variation between homologs (called “degenerate primers”). For this reason, Apprill et al. (2015) used primer 515F and 806RB with degeneracy to reduce bias and, consequently, resolve the underestimation of the SAR11 clade in marine samples. Walters et al. (2016) compared the performance of the original 806R primer and the 806RB degenerate primer for detecting the SAR11 clade (a ubiquitous and abundant marine bacterial group) and observed that the degenerate primer not only increased the detection of the SAR11 clade but also interfered with the performance of taxa amplified by the original primer.

Taq polymerase – Taq polymerase is a thermostable enzyme that can synthesize DNA only when given a primer that provides a starting point for synthesizing a DNA region of interest. There are different types of Taq polymerase with each having a unique fidelity (i.e., accuracy) and processivity (i.e., how quickly it synthesizes) that can help you choose the most appropriate Taq polymerase for your study. Due to the diversity of Taq polymerase brands, there is no single Taq polymerase that is best suited for every study. Instead, the preferences of some researchers depends on the sample type, the efficiency of the Taq polymerase, the cost, and the practicality of use. As an enzyme, Taq polymerase requires the presence of a cofactor during the PCR reaction, such as Mg^2+^ ions. Some manufacturers offer Taq polymerase in a buffer containing this cofactor at a standard concentration, but others provide it as an aside or as an addition. However, magnesium chloride (MgCl_2_; Markoulatos et al., 2002) if used in high concentrations, can lower the specificity of Taq and create spurious primer pairings (i.e., matches between primers and unwanted sites in the template DNA). Not only can excessive addition of Mg^2+^ cause problems during the action of Taq polymerase, but some inhibitors that come from DNA extraction or poor DNA purification can directly affect Taq polymerase. These inhibitors can prevent the interaction of Taq with Mg^2+^ ions (e.g., Ca^+^ ions from the skeleton), thereby preventing the action of Taq polymerase in the DNA amplification process. Furthermore, other contaminants can interact directly with Mg^2+^ ions, reducing their concentration and preventing the catalytic action with Taq polymerase.

dNTPs (deoxynucleotides 5′-triphosphates) – dNTPs are used in PCR to provide nucleotides that will be added to the growing oligonucleotide chain during the synthesis of new DNA amplicons (Markoulatos et al., 2002; Paul and Yee, 2010). Some manufacturers will add dNTPs to a buffer that includes both the Taq polymerase and Mg^2+^ ions in effective ratios, while others will provide them as an aside. If adding dNTPs separately, it is important to note that high concentrations can chelate Mg^2+^ ions reducing the effective function of Taq polymerase (Roux, 1995); it is thus necessary to work with small volume aliquots so that there is no loss of oligonucleotide yield.

DNA template – The purity of the DNA in the PCR technique is essential for accurate and effective microbiome analyses to be carried out. Thus, the DNA sample must be free of any inhibitors (see section on DNA extraction) and free of exogenous or contaminant DNA (see below for discussion). To check for inhibitors, DNA quantification performed by UV spectrophotometer can differentiate DNA from inhibitors through wavelength analysis (Boesenberg-Smith et al., 2012). In addition, an excessive amount of DNA can inhibit the amplification process.

#### 3.3.1. Coral host off-target PCR contamination

Another major challenge in coral microbiome work is the efficient amplification of ‘off-target’ coral DNA sequences alongside microbial genes. In many coral species, several popular primers used for 16S rRNA amplification (e.g., 515F-806RB) have high similarity to coral mitochondria and chloroplast genes due to their shared ancestry with bacteria (Lopez et al., 2003). Non-specific or off-target amplification of coral host DNA can create multiple PCR products that result in a pool of eukaryotic amplicons mixed with bacterial amplicons (Galkiewicz and Kellogg, 2008). Without separation, the resulting libraries will contain both amplicons and reduce the sequencing depth of the target amplicon, potentially leading to an underestimate of the true diversity and/or taxonomic profile of the microbial community.

**Figure 2:**
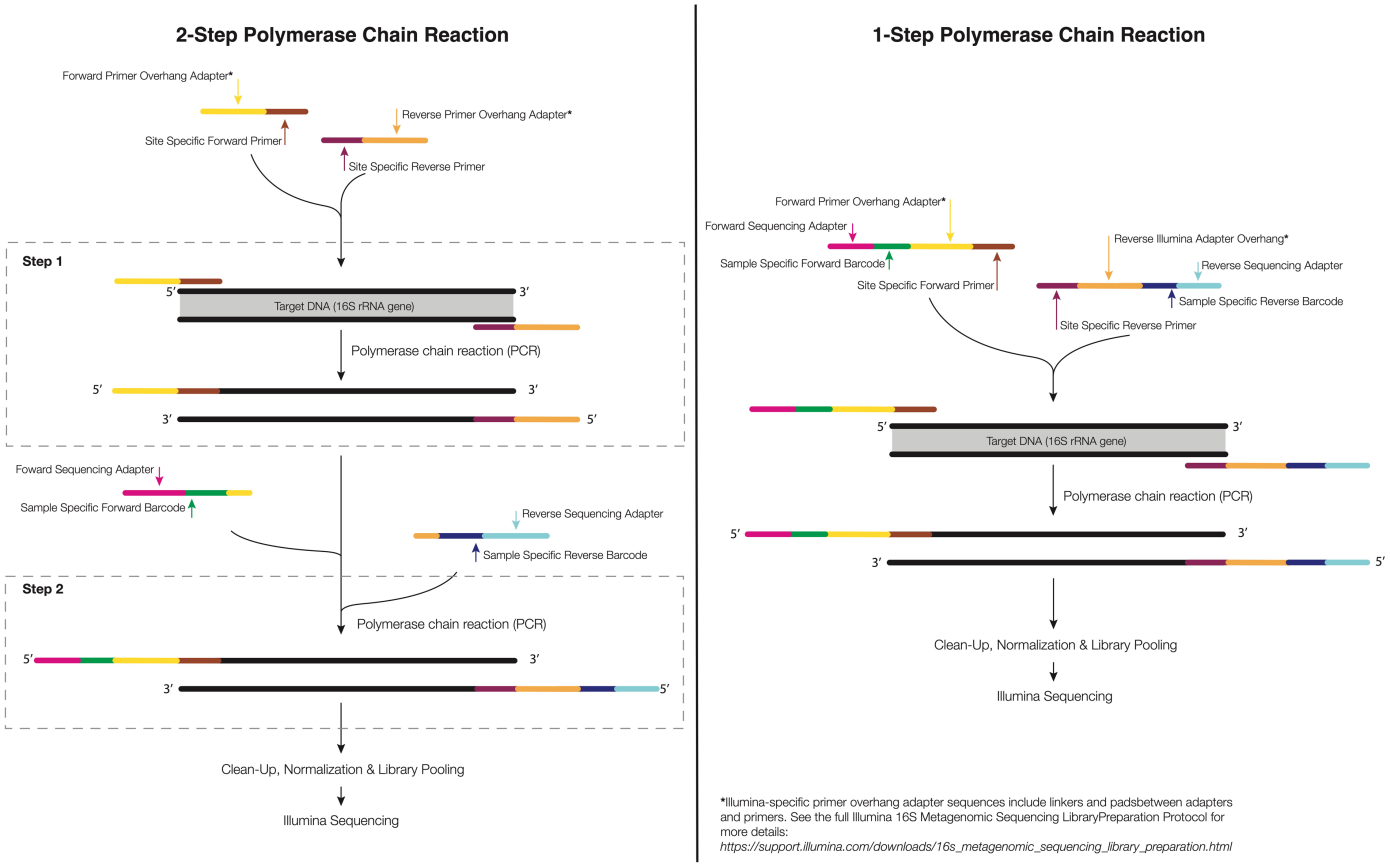
1-step and 2-step PCR amplification approach. In the 1-step PCR (right), the target gene is amplified using primers sequence composed of forward and reverse target gene primer, overhang linkers, barcodes, and Illumina adapters to bind to a flow cell in Illumina sequencing. In the 2-step PCR amplification (left), the primer of the first-step PCR contains a specific forward and reverse primer and an overhang adapter. For the second-step PCR, the primers have an overhang linker, barcodes, and the Illumina flow-cell linker sequence.

Steps to minimize, eliminate, or sidestep the off-target amplification issue are available, however. As eukaryotic DNA becomes available during the cell lysis step of DNA extraction, downstream optimization of the PCR protocol or purification methods can be used to minimize the amplification of non-microbial DNA during PCR. For example, Galkiewicz and Kellogg (2008) used an alternative primer set (63F/1542R) to separate eukaryotic from bacterial rRNA genes during the PCR technique. However, according to the authors, care should be taken when selecting the 63F primer, as this can develop a bias in the bacterial profile generated. Ten years later, Pollock et al. (2018) reported that primers that amplify the V4 region of the 16S rRNA, 515F-806R, also amplify the mitochondrial 12S rRNA gene from coral. However, these amplicons are slightly different lengths and can be removed or annotated separately *in silico*. The 16S rRNA gene amplicon is shorter ~300 bp, while the 12S rRNA off target coral gene amplicon is longer ~400 bp. As such, off target amplicons can be removed *via* gel-based size selection purification methods (e.g., using a BluePipin machine) and/or a 2-step PCR where only the proper size band is excised and barcoding is conducted on exclusively the targeted 16S band (see Figure 2 for details). Explicitly, after the first PCR with only the locus-specific primers, agarose gel electrophoresis is applied for the separation of the 12S and 16S amplicons. Given the band sizes are similar this can require a slow and long gel separation step. Next, the 16S rRNA amplicons are chemically purified (i.e, PCR clean up kits) or physically removed (i.e., excised with a sterile tip or razor blade) from the electrophoresis gel, and used for the DNA template in a second step of PCR (Caporaso et al., 2011). This technique is efficient and, when conducted properly, can eliminate a majority of the off-target amplicon. It is possible to also use peptide nucleic acid (PNA) clamps to bind to target DNA, preventing host DNA amplification and increasing bacterial DNA amplification (Reigel et al., 2020). This can provide a cheap and efficient alternative method for the decontamination of microbial DNA without underestimating the rare biosphere.

### 3.4. HTS library construction

For sequencing to be successful, it is necessary to prepare individually identifiable ‘sequencing libraries’ for each coral microbiome sample. As HTS platforms sequence many samples simultaneously (‘multiplex’), each coral microbiome library must contain a unique coded set of nucleic acid markers or ‘barcodes’ that indicate which sample is which within the final pool that is sequenced. Barcodes are small oligonucleotide sequences (usually 8–12 nucleotides in length) used to identify sequences from a given sample that allows the pooling, or multiplexing, of several samples into a single library that can be sequenced on a single sequencing lane or run (Head et al., 2014; Lebonah and Chandrasekhar, 2014; Zhang et al., 2020). At the same time, individually barcoded samples must be purified, ensuring that they are free of extraneous nucleic acids including any remaining forward and reverse primers and/or primer dimers.

The complete process can require a different number of steps depending on whether one is conducting 1 or 2 step PCR (Figure 2; see above discussion on host 12S contamination). A 1-step protocol includes attaching specific forward and reverse primers containing the 16S rRNA region, and a sequence tail called overhang linker sequences, barcodes and adapters to allow binding to a flow cell in Illumina sequencing (currently, the most used sequencing; see Figure 2). In the 2-step protocol, the same initial PCR step is completed including specific forward and reverse primers, and overhang linker sequence. The second PCR step includes a small overhang linker sequence, barcodes, and Illumina adapters (Figure 2). The advantage of a 2-step PCR is the flexibility to amplify the gene target of low-biomass samples when compared with the 1-step PCR as well as the ability to ensure off-target sequences are avoided. However, the downside is that an additional PCR step must be completed which can increase the financial and opportunity cost of library generation. Further care must be taken when conducting multiple step PCR as any additional rounds of amplification can increase the risk of producing artifacts (Kozich et al., 2013).

For corals, library preparation can also be 1 or 2 steps (Figure 2). Two step PCR approaches generally require: (1) PCR for the separation of the 12S rRNA genes from the 16S rRNA and (2) validation by gel electrophoresis (a 1% agarose). The 16S rRNA amplicons derived from the electrophoresis gel must be purified or the reaction used as the target DNA to perform the second-step PCR where the indices/barcodes are added (Figure 2).

After amplification, amplicons must undergo purification to build a refined library since sequencing is a highly sensitive technique. At this stage, a more efficient method of purification is used, such as the use of magnetic beads in which the amplicons bind reversibly and undergo a simple washing process to remove the primers, primer-dimers, nucleotides, salts, and enzymes (Watson and Blackwell, 2000). An elution reagent (e.g., TE Buffer) or nuclease-free water is used to elute the amplicons for a purified final product. There are some commercially available library preparation kits that can streamline this process, such as Illumina DNA Prep and TruSeq DNA PCR free. Many perform purification by eliminating both short and long fragments through a two-step process. The long fragments first bind to the magnetic beads, then the supernatant is removed and purified to remove the short fragments. During bead purification, it is possible to size select the amplicons based on the proportion of beads to a sample volume. Most commercial kits are designed to capture amplicons >100 bp and eliminate <50 bp, but these values can be changed according to the size of the library of interest. It is important to note that the proportion of beads to sample can affect the final library and performance.

#### 3.4.1. Quantifying and combining amplicons for multiplexed library sequencing

**Figure 3:**
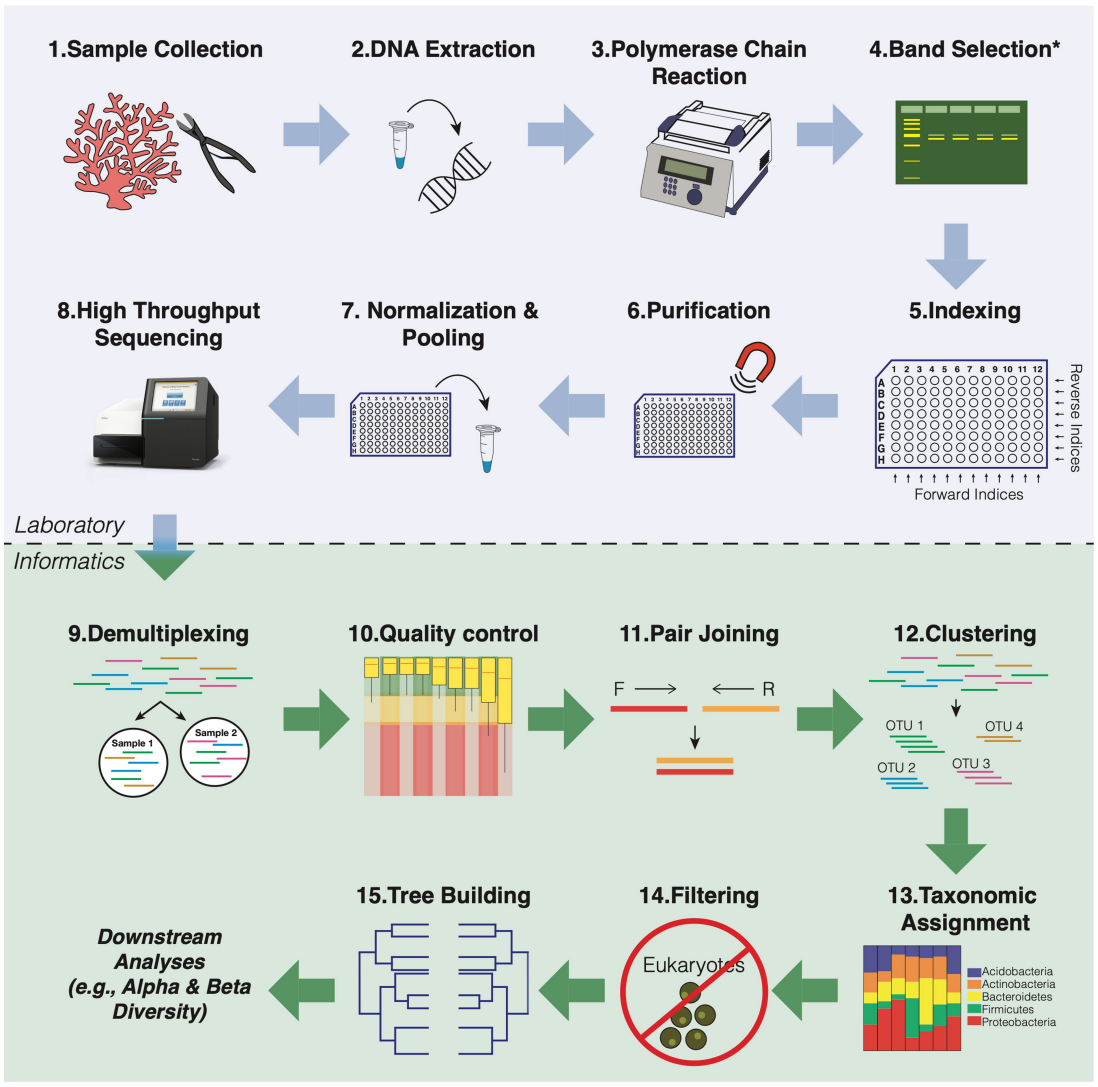
General workflow overview for 16S rRNA amplicon sequencing of coral microbial communities. The blue box indicates all the steps for preparing the library. The green box shows all the steps of bioinformatic data analysis that refer to the preparation of the readings for the downstream analyses.

After purification of the amplicons, libraries must be quantified and mixed in similar proportions, so that the library will be equally represented in the final pool. Otherwise, samples that are amplified better than others may be over-represented in the dataset while others will have read levels so low that they cannot be used in the final analysis. Quantification and sizing of the gene library are performed by spectrophotometry (e.g., UV/Vis or Nanodrop), fluorometry (Qubit, Picogreen), quantitative PCR (qPCR), or droplet digital PCR (ddPCR). However, at this stage, care must be taken when using spectrophotometric quantification, as any impurity present can contribute to the absorbance. For the final library step, amplicons should be pooled at similar molar concentrations. Typically, an optimal initial library concentration is at least 4 nM (Illumina, 2019). It is noteworthy that libraries with values less than 1 nM will have very low yields. The quality of the final library can be checked on an agarose gel or more accurately using a Bioanalyzer (e.g., Agilent 2,100). A workflow for HTS library preparation is shown in Figure 3, including all the steps mentioned above.

### 3.5. High-throughput sequencing steps

In preparation for cluster generation and sequencing on the standard Miseq sequencing platform, double-stranded libraries are denatured using sodium hydroxide (NaOH) in concentrations between 0.1–0.2 N, respectively, for samples with amplicon concentrations between 0.5 and 4 nM. High concentrations of NaOH can inhibit the hybridization of the library in the flow cell and, thus, decrease the cluster density (Wu et al., 2018a). Then, amplicons are diluted with an HT1 buffer (hybridization buffer) at the picomolar level l for final loading into flow cells.

It is noteworthy that some biases can affect the construction of a refined library, such as cross-contamination of indexed primers producing chimeras by recombination of different molecules (Kircher et al., 2012). Also, in 16S rRNA and almost all other amplicon libraries, sequences exhibit low base diversity, or an imbalance in the number and order of bases in a set of sequences. This imbalance can negatively impact the cluster model formed during sequencing. To expand the diversity and enrich this library with unique sequences, it is recommended to add a shotgun library to the pool. Typically, this is a PhiX library (the genome of the ΦX174 bacteriophage that is cut into small random segments) in Illumina sequencing. The phiX library is a ready-made library that provides quality control for the alignment and sequencing of clusters due to its diverse composition of bases (45% GC and 55% AT) and can be applied to increase confidence in your results. The concentration of PhiX to be added will depend on fragment length and sequencer software (Kozich et al., 2013). In some instances, you may be able to provide DNA from a diverse sample of your own (e.g., a coral microbiome sample) in place of PhiX, which can generate between 2 and 12 million bases of metagenome that may be used in downstream metagenomic analyses. The use of a PhiX replacement should be discussed with your sequence provider.

## 4. Considerations for bioinformatics

The sequencing analysis of 16S rRNA amplicons is based on software and algorithms that convert this sequencing data into biologically meaningful results. Bioinformatic pipelines from a variety of software programs can quickly and efficiently perform these analyses. While most software programs and pipelines include a similar sequence of steps for denoising, merging, grouping and taxonomy assignment to 16S rRNA sequences, they can vary by quality control parameters and clustering algorithms. Commonly used software programs to build 16S bioinformatics pipelines include Quantitative Insights into Microbial Ecology 2 (QIIME 2; Bolyen et al., 2019) with options for DADA2 or Deblur ASV-picking algorithms, mothur (Schloss et al., 2009; Schloss, 2020), DADA2 (Callahan et al., 2016) in R (Team R.C, 2020), and USEARCH (including UPARSE and UNOISE; Edgar, 2010). Although USEARCH is widely used, it is not open-source software and therefore has limitations for its use and redistribution. VSEARCH (Rognes et al., 2016) can be used as an open-source alternative to USEARCH.

These software programs have options for analysis at both the OTU (Operational Taxonomic Unit) and ASV (Amplicon Sequence Variant) levels (detailed in the Merge reads and Clustering section below). Previous studies have examined the sensitivity and consensus differences in several of these pipelines using default settings to mimic what most users have likely implemented (see Plummer et al., 2015; Prodan et al., 2020); however, customization can improve the performance of any pipeline. Below is a summary of each step in the 16S rRNA bioinformatic pipeline using the most widely used bioinformatic tools in coral microbiome studies, including a discussion on the differences between pipelines. The bioinformatics analysis steps used to process the data are cited below and shown in Figure 3.

### 4.1. Demultiplexing

Demultiplexing is the first ‘*in silico*’ step after sequencing, in which the barcode sequences are used to identify and group sequences that come from the same sample. In some cases, the sequence provider will complete this step prior to returning sequence data to the user given that a spreadsheet identifying the sample barcodes is provided. When samples are returned multiplexed, demultiplexing can be done using most bioinformatic pipelines. QIIME 2 uses the “q2-demux” plug-in that can demultiplex both single and paired-end sequence reads. The barcodes are read as a reverse complement of the original sequence through the script “-p-rev-comp-mapping-barcodes” in the demultiplexing of paired readings. If adapters and primers are still present on the sequences, a cutadapt plug-in (Martin, 2011) for QIIME2 called “q2-cutadapt” can be used. Mothur uses a command called “make.contigs” for demultiplexing, where paired-end reads are also merged at the same time. This mothur command has the option to add an “oligos” parameter for removing primers and barcodes, and a “check orient” parameter to search for the reverse complements when primer and barcode sequences cannot be found.

While both QIIME2 and mothur have the ability to perform demultiplexing, OTU and ASV picking requires the input sequence data to be demultiplexed and trimmed (adapters, primers, and barcodes removed). This can be done using other pipelines or software (e.g., cutadapt, trimmomatic; Bolger et al., 2014) or even command line computation (e.g., using Python or Biopython).

### 4.2. Quality control

It is essential to check the quality of the sequences to avoid overestimating microbial diversity. Quality filtering is often used to truncate or discard overlapping matched reads to minimize the presence of any sequencing errors. The accuracy of sequencing is assessed by the Phred quality score (Q-score) provided for each nucleotide, which indicates the probability of an incorrect base call (Nilakanta et al., 2014); the higher the Q-score, the lower the probability of an incorrect base call. Pipelines such as QIIME2, DADA2, and other standalone software, such as FastQC (Wingett and Andrews, 2018), have a graphical user interface, which can visualize the quality scores for either the entire library or each forward and reverse read. These graphs can be used to set the parameters for trimming and noise reduction. It is worth mentioning that these parameters will differ for each dataset and read direction, as reverse reads are often of lower quality than forward reads, and should be optimized to avoid data loss through excessive reduction of the read length. Parameters for quality filtering include (1) primer removal, (2) off target outlier sequence read removal, (3) removal of poor-quality reads with Phred scores <4 and >60, and (5) removal of any reads that exceed a defined maximum number of “expected errors” (maxEE). QIIME2 incorporates either DADA2’s quality control steps through the “dada2” plug-in, using the”dada2 denoise” command with the parameters “--p-trim-left” and “--p-trunc-len,” or with Deblur through the “deblur denoise-16S” command with the parameter “--p-trim-length.” In mothur, quality filtering is performed using the “screen.seqs” command. In DADA2, the filtering is done through the command “filterAndTrim.” Both commands allow parameters to be defined for each forward and reverse read.

### 4.3. Merge reads and clustering

Clustering sequences based on similarity allows for accurate downstream identification of putative microbial species for taxonomic assignment and statistical analysis. For paired end sequencing, merging forward and reverse reads must occur prior to clustering, and is often incorporated into the clustering commands for simplification of the pipeline. There are a number of different sequence similarity thresholds that have been used, and the choice for which threshold to pick should be dependent on the biological or ecological question that is posed. Sequences with a 97% or greater similarity can be grouped into what are called operational taxonomic units (OTUs). Historically, OTUs have been treated as similar to observing a species (Callahan et al., 2017). As sequencing technologies and bioinformatic analyses have improved our ability to identify sequencing errors, we can now group sequences based on higher thresholds, such as 99% or 100%, that represent amplicon sequence variants (ASVs), oligotypes, exact sequence variants (ESVs), or zero radius operational taxonomic units (zOTUs). Clustering methods based on higher thresholds, such as ASVs, infer biological sequences before amplification and sequencing errors and distinguish variants by only 1 nucleotide (Callahan et al., 2017). Clustering can be performed “*de novo*” without reference sequences, which creates the sequence clusters only through observed similarity and not based on a database. In contrast, closed reference clustering methods require a reference database to compare to the observed sequences, and any sequences that are not present in the reference databases may be lost. QIIME 2 provides two options for *de novo* ASV-picking: DADA2 using the plug-in “q2-dada2” (Callahan et al., 2016) or Deblur using the plug-in “q2-deblur” (Amir et al., 2017). With both methods in QIIME2, the joining of paired reads will be performed automatically during denoising. OTU-picking in QIIME2 utilizes VSEARCH *via* the “q2-vsearch” plug-in (Rognes et al., 2016). In DADA2, the readings are duplicated, and the ASVs inferred. In this case, DADA2 works by retaining a summary of quality scores associated with each sequence and thus performs the inference of ASVs. Then the forward and reverse ASVs are merged using the “mergepairs” command. In mothur, sequences are assigned to OTUs *via* the “Cluster” command. This command can be based on different clustering methods, including Search (does not require distance matrix), but commonly used methods are based on percentage distance between sequences. Furthermore, ASVs can be identified in mothur through the “pre-cluster” command.

### 4.4. Taxonomic assignment

Once sequences have been clustered, taxonomy can then be assigned. The taxonomic nomenclature is based on reference databases, of which the most popular in 16S rRNA-based phylogeny analysis include SILVA (Pruesse et al., 2007; Yilmaz et al., 2013), Greengenes (McDonald et al., 2012) and the Ribosomal Database Project (RDP; Wang et al., 2007). One of the main differences between these taxonomic databases is the origin of taxonomic rank information. For instance, the taxonomic classification for the RDP is obtained from the International Nucleotide Sequence Database Collaboration (INSDC). SILVA is based on Bergey’s Taxonomic Outlines, List of Prokaryotic Names with Standing in Nomenclature (LPSN) and is manually curated. Greengenes is based on the National Center for Biotechnology Information (NCBI). It is important to note that taxonomic compositions of a dataset will depend on which taxonomic reference database is used (Sierra et al., 2020). Most bioinformatic pipelines offer a default taxonomic classifier; mothur uses RDP and QIIME2 uses Greengenes. However, these can be manually replaced by any other database. The algorithm for taxonomic classification can also differ. For instance, both QIIME 2 and DADA2 use a naïve Bayesian trained classifier, where the classifier is first trained on the specific region of the target sequences. These taxonomic classifiers are prepared based on specific sequencing parameters and target sequence compliance, which creates a new, dataset-specific taxonomic attribution repository.

### 4.5. Removal of unwanted taxa

After taxonomic assignment, a filtering step can be performed to remove any unwanted taxa (e.g., any eukaryotic contamination) or optimize the feature table. In QIIME2, some parameters can be optimized in this step, such as removing ASVs present only in 1 sample that may not represent the true biological diversity (perhaps errors during PCR amplification and sequencing). In addition, libraries can be curated to contain a minimum total number of reads to normalize the analysis across the datasets. This process called ‘rarefaction’ is however controversial. For additional reading on this topic see the works by Hughes and Hellmann (2005) and Willis (2019). It is noteworthy that even after the separation of 12S rRNA host during 2 step PCR, we often still find reads from the host, making the removal of chloroplasts and mitochondria from taxonomic attributions a critical step before undertaking downstream analyses. A supplementary database called Metaxa2 (Bengtsson-Palme et al., 2015) was created to assess the effects of mitochondrial sequences in the analysis of bacterial diversity and this database might underreport the existence of mitochondrial sequences in coral microbiome samples. That databases might underreport the existence of mitochondrial sequences in coral microbiome samples. The inclusion of mitochondrial reference sequence databases such as Metaxa2 is recommended for coral microbiome samples.

In QIIME 2, the removal of chimeras, where two or more sequences have been incorrectly joined together during sequencing, should also be done. This filtering is performed by the plug-in “q2-feature-table”. In mothur, mitochondria exclusion and the removal of chimeras are performed before taxonomic attribution through the command “remove.lineage”: and “chimera.Vsearch.” In DADA2, the removal of chimeras is done with the function “removeBimeraDenovo.”

## 5. Downstream analysis

**Figure 4:**
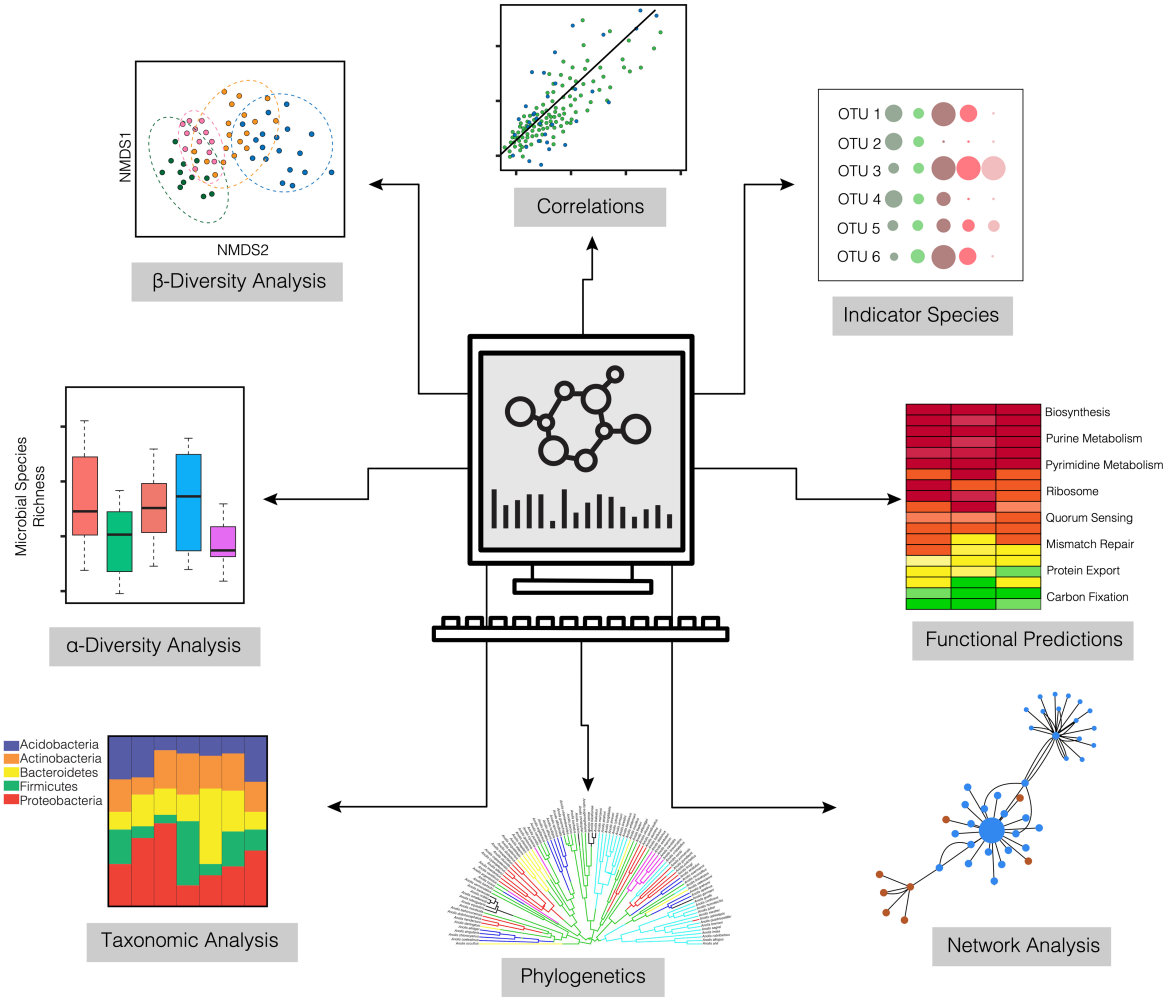
Downstream analyses for characterizing the abundance, diversity, and composition of coral microbiomes derived from 16S amplicon sequencing.

After the computational treatment of the OTUs/ASVs, output files are generated that are available for taxonomic analysis, alpha and beta diversity estimation, measurement of dispersion, and even estimates of functional pathways. Output files generated by bioinformatic pipelines that are necessary for downstream statistical analyses include an OTU or ASV feature table of raw sequence counts (biom format file), a taxonomic reference file for each OTU or ASV (csv or txt format), and a phylogenetic tree file (newick format). With these data in hand, researchers can begin to unravel the microbiome dynamics of their individual systems. There is a diverse range of analytical and statistical tests that can be explored for 16S rRNA amplicon data that are not discussed in depth in this paper (Figure 4). These include, but are not limited to, functional prediction based on taxonomy (e.g., Picrust2; Douglas et al., 2020), network analyses that infer community co-occurrence (Barberán et al., 2012), and multi-level pattern analyses that can identify bacterial indicator species (Dufrêne and Legendre, 1997).

### 5.1. Alpha and beta diversity metrics

Many ecological paradigms (e.g., resistance, resilience, and stable state dynamics) are built around how diversity changes in response to a given disturbance. Yet metrics of biodiversity come in many forms; knowing the difference among these is critical to understanding biological patterns in a study system. Alpha diversity is a collection of measures that characterize several aspects of the number of different taxa and their uniformity in a community. Alpha diversity can include metrics such as ‘species’ richness (i.e., the exact observed number of OTUs or ASVs of a given taxon), Chao1 (predicted richness based on species accumulation curves), evenness (the numerical distribution of different taxa relative to one another within a community), Shannon Index (an index that incorporates aspects of both richness and evenness), and inverse Simpson index (value between 0 and 1 represents the increase in diversity based on the average proportional abundance and the number of species). These metrics can be visually expressed, for example, through rarefaction curves. Furthermore, scatter plots of alpha diversity metrics against environmental measures (e.g., temperature, pH, dissolved oxygen, nutrients) can provide important insights into drivers of diversity within an ecosystem. These relationships can be tested using univariate statistical models, such as least-squares regression.

Beta diversity measures, unlike alpha diversity, assess the variety and relative abundance of different species that make up the microbiome. Beta-diversity is typically reported as either between variable beta-diversity or within variable beta-diversity, a measure also referred to as ‘dispersion’ which we discuss below. Beta-diversity measures are usually constructed from matrices that include all the taxa and their comparative abundances. Differences among samples or locations based on a given variable (e.g., host species, sampling time points, some experimental or environmentally altered variable like temperature) are typically tested through permutational multivariate analyses (e.g., PERMANOVA) and visualized using ordination methods such as non-metric multidimensional scaling (NMDS), Principal Coordinate Analysis (PCoA), or Canonical Correspondence Analysis (CCA).

Differences in diversity between samples can provide insights into how microbiomes may change through time, between host health states, or among any target variable. These changes can be either deterministic (shift in the same way) or stochastic (shift in different ways). It has been suggested in the literature that environmental and health stressors of corals cause microbiome destabilization that is represented by stochastic changes in microbial community structure (see Zaneveld et al., 2016) and can be visualized by dispersion effects in ordination space (i.e., how close microbiomes of different samples cluster). As a result, highly dispersed microbiomes have been associated with negative impacts such as disease (e.g., Rosales et al., 2019; Becker et al., 2022) and anthropogenic disturbance (e.g., Zaneveld et al., 2016; Maher et al., 2019).

Different methods can estimate microbial community variances, such as methods based on dispersion estimation of individual taxa or communities of taxa using a weighted conditional probability [e.g., EdgeR see (Robinson and Smyth, 2007; Chen and McCarthy, 2015)]; or methods that model the dispersion of individual taxa by averaging the heterogeneity of the dispersion values for different taxa using a Bayesian approach (e.g., DSS, see Wu et al., 2013). We recommend the use and exploration of various multivariate analysis techniques that are required by the multidimensional nature of microbiome community data.

### 5.2. Differential abundance analysis

Depending on the research question and/or experimental design, it is commonly of interest to determine how the abundance of certain microbes varies among treatments or environments. These differentially abundant taxa may represent important biomarkers for coral health or other factors that may impact coral resilience. Multiple types of differential abundance (DA) analytical tools exist for use in microbiome studies, including traditional statistical tests (e.g., t-tests or Kruskal-Wallis rank sum tests), those originally designed for differential gene expression [e.g., DESeq2 (Love et al., 2014)], and those developed specifically for microbiome studies [e.g., Analysis of Composition of Microbiomes (ANCOM; Mandal et al., 2015)] and ANCOM with Bias Correction (Lin and Peddada, 2020). However, many of these tools face challenges associated with the treatment of microbial count data (see Swift et al., 2022), making this an active area of method development and care should be taken when choosing the appropriate test for your data.

## 6. Conclusion

Here, we reviewed current methods to sample, extract, and analyze the coral microbiome based on 16S rRNA gene sequencing. Coral represents a complex animal-symbiont holobiont whose molecular and *in silico* 16S rRNA pipelines may require technical adaptations that, in some cases, may differ in methodology from those described in manufacturer’s documents or in the literature for more simple or well-studied host systems. Furthermore, careful consideration must be made of appropriate methods that meet study objectives while also accounting for differences in methods required for the various compartments and needs specific to different corals. Nevertheless, with an expanded number of field, laboratory, and computer techniques and tools available, reduced costs of analysis, and increased applicability, conducting coral microbiome research is increasingly available to new and established investigators. Due to the complexity of bioinformatic methods, the sections describing HTS and considerations represent a basic starting point for those pursuing 16S rRNA amplified sequence studies. However, more in-depth reading of the subject is recommended according to one’s study aims. We hope that this review provides a condensed platform of knowledge and a set of methodologies to those initiating research in this area. Together we can advance and accelerate coral microbiome research and ideally the management and conservation of coral reefs worldwide.

## Author contributions

DS and RVT conceptualized the manuscript. DS collected literature data and wrote the first draft of the manuscript. DS, RVT, and HE wrote text, provided revisions, created figures and editing to the manuscript. All authors contributed to the article and approved the submitted version.

## Funding

Financial support from National Science Foundation (NSF) Rules of Life: Microbiome Grant number 2025457 to RVT and a NSF PFRB Award #2006244 to HE.

## Conflict of interest

The authors declare that the research was conducted in the absence of any commercial or financial relationships that could be construed as a potential conflict of interest.

## Publisher’s note

All claims expressed in this article are solely those of the authors and do not necessarily represent those of their affiliated organizations, or those of the publisher, the editors and the reviewers. Any product that may be evaluated in this article, or claim that may be made by its manufacturer, is not guaranteed or endorsed by the publisher.
